# PIMR: Parallel and Integrated Matching for Raw Data

**DOI:** 10.3390/s16010054

**Published:** 2016-01-02

**Authors:** Zhenghao Li, Junying Yang, Jiaduo Zhao, Peng Han, Zhi Chai

**Affiliations:** 1 Key Laboratory for Optoelectronic Technology and Systems of Ministry of Education, College of Optoelectronic Engineering, Chongqing University, Chongqing 400044, China; yangjunying_90@cqu.edu.cn (J.Y.); jdzhao@cqu.edu.cn (J.Z.); 2 Chongqing Academy of Science and Technology, Chongqing 401123, China; hanpeng@cqu.edu.cn; 3 Beijing Institute of Environmental Features, Beijing 100854, China; ezhchai@163.com

**Keywords:** image sensor, raw data, image matching, image analysis

## Abstract

With the trend of high-resolution imaging, computational costs of image matching have substantially increased. In order to find the compromise between accuracy and computation in real-time applications, we bring forward a fast and robust matching algorithm, named parallel and integrated matching for raw data (PIMR). This algorithm not only effectively utilizes the color information of raw data, but also designs a parallel and integrated framework to shorten the time-cost in the demosaicing stage. Experiments show that compared to existing state-of-the-art methods, the proposed algorithm yields a comparable recognition rate, while the total time-cost of imaging and matching is significantly reduced.

## 1. Introduction

Image matching is a crucial technique with many practical applications in computer vision, including panorama stitching [1], remote sensing [2], intelligent video surveillance [3] and pathological disease detection [4].

Hu’s moment invariants as a shape feature has been widely used for image description due to its scaling and rotation invariance [5]. Wang *et al*. further proposed a two-step approach used for pathological brain detection by employing this feature combined with wavelet entropy [6]. Lowe’s scale-invariant feature transform (SIFT) is a *de facto* standard for matching, on account of its excellent performance, which is invariant to a variety of common image transformations [7]. Bay’s speeded up robust features (SURF) is another outstanding method performing approximately as well as SIFT with lower computational cost [8]. We proposed a lightweight approach with the name of region-restricted rapid keypoint registration (R^3^KR), which makes use of a 12-dimensional orientation descriptor and a two-stage strategy to further reduce the computational cost [9]. However, it is still computationally expensive for real-time applications.

Recently, many efforts have been made to enhance the efficiency of matching by employing binary descriptors instead of floating-point ones. Binary robust independent elementary features (BRIEF) is a representative example which directly computes the descriptor bit-stream quite fast, based on simple intensity difference tests in a smoothed patch [10]. When combined with a fast keypoint detector, such as features from accelerated segment test (FAST) [11] or center surround extrema (CenSurE) [12], the method provides a better alternative for real-time applications. Despite the efficiency and robustness to image blur and illumination change, the approach is very sensitive to rotation and scale changes. Rublee *et al*. further proposed oriented FAST and rotated BRIEF (ORB) on the basis of BRIEF [13]. The approach acquires multi-scale FAST keypoints using a pyramid scheme, and computes the orientation of the keypoints utilizing intensity centroid [14]; thus, the descriptor is rotation- and scale-invariant. In addition, it also uses a learning method to obtain binary tests with lower correlation, so that the descriptor becomes more discriminative accordingly. Some researchers also try to increase the robustness of matching by improving the sampling pattern for descriptors. The binary robust invariant scalable keypoints (BRISK) method proposed by Leutenegger adopts a circular pattern with 60 sampling points, of which the long-distance pairs are used for computing the orientation and the short-distance ones for building descriptors [15]. Alahi’s fast retina keypoint (FREAK) is another typical one leveraging a novel retina sampling pattern inspired by the human visual system [16]. Leutenegger uses a scale-space FAST-based detector in BRISK to cope with the scale invariance, which is employed by FREAK as well.

However, several inherent problems remain in existing methods. The digital image which consists of 24 bit blue/green/red (BGR) data is color-interpolated, which is known as demosaicing from raw data, including adjustment for saturation, sharpness and contrast, and sometimes compression for transmission [17,18]. This operation leads to irreversible information loss and quality degeneration. Furthermore, up until now, conventional image matching has been implemented after demosaicing, and such sequential operation restricts its application to general tasks. To address these limitations, in this paper we introduce an ultra-fast and robust algorithm, coined parallel and integrated matching for raw data (PIMR). The approach takes raw data instead of a digital image as the object for analysis, which is efficient for preventing information from being tampered with artificially. It is crucial to obtain high-quality features with the result that the approach can achieve comparable precision. Meanwhile, a parallel and integrated framework is employed to accelerate the entire image matching, in which two cores are used to respectively process the matching and demosaicing stages in parallel. Our experiments demonstrate that the proposed method can acquire more robust matches in most cases, even though it is much less time-consuming than traditional sequential image matching algorithms, such as BRIEF, ORB, BRISK and FREAK.

The rest of the paper is organized as follows. Section 2 gives the implementation details of the proposed method, which mainly includes the raw data reconstruction, and the parallel and integrated framework. In Section 3, we evaluate the performance of PIMR. Lastly, in Section 4, conclusions are presented.

## 2. Parallel and Integrated Matching for Raw Data

In Section 2.1, we give a brief account of raw data to make the reader understand our work more clearly. The key steps in PIMR are explained in Section 2.2 and Section 2.3, namely the reconstruction step for raw data and the details of the parallel and integrated framework.

### 2.1. Raw Data

Raw data, which is the unprocessed digital output of an image sensor, represents an amount of electrical charges accumulated in each photographic unit of the sensor. The notable features of raw data are nondestructive white balance, lossless compression, and high bit depth (e.g., 16 bits), providing considerably wider dynamic range than the JPEG file [19,20,21]. Hence, raw data maximally retains the real information of the scene compared to the digital image. By directly analyzing raw data, not only does it prevent the original information from being tampered with artificially, which contributes to an increase in precision, but it also shortens the time-cost in the demosaicing part.

**Figure 1:**
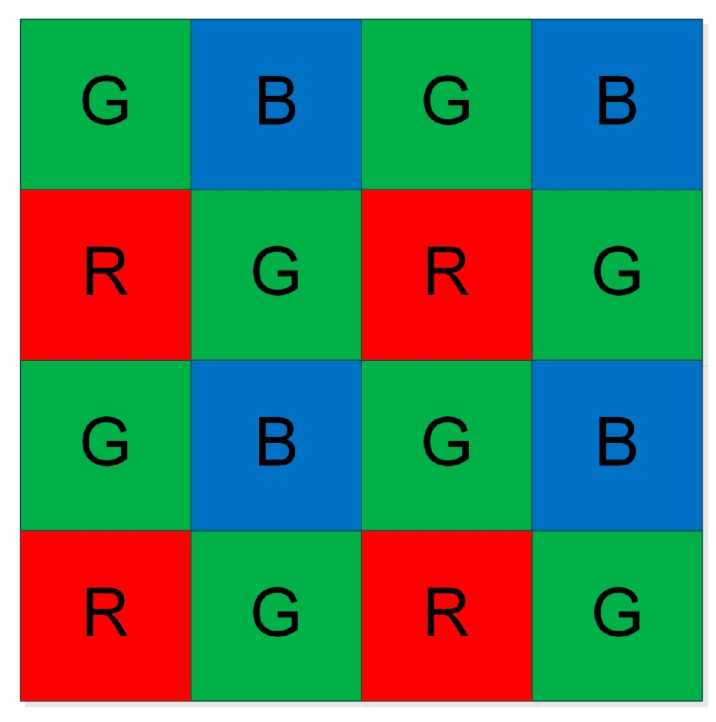
Bayer pattern of GBRG mode.

The most popular format of raw data is Bayer Tile Pattern [22,23], typically in GBRG mode. It is widely used in industrial digital cameras. As illustrated in Figure 1, it is basically a series of band pass filters that only allow certain colors of light through, which are red (R), green (G) and blue (B). Green detail, to which the human visual system is more responsive than blue and red, is sampled more frequently, and the number of G is twice that of B and R.

### 2.2. Reconstruction for Raw Data

At each pixel location, the sensor measures either the red, green, or blue value, and the values of other two are highly related to the neighbors around the pixel. On this basis, in order to make raw data more appropriate for further processing, we reconstruct it as follows. Initially, we define the 2 × 2 set of pixels as a cell. It starts with the target pixel *C*(*i*, *j*), and the other three pixels belonging to the cell are *C*(*i* + 1, *j*), *C*(*i*, *j* + 1) and *C*(*i* + 1, *j* + 1), respectively, where *C* represents an arbitrary color in *G*, *B* and *R*. Next, we utilize the color information in each cell to reconstruct the intensity *I* for the target pixel:

(1) $$I_{\text{MAX}}=\max(G+G^{\prime },B+R)$$

(2) $$I_{\text{MIN}}=\min(G+G^{\prime },B+R)$$

(3) $$I=(w_{1}I_{\text{MAX}}+w_{2}I_{\text{MIN}})/2$$

where *G*, *G*’, *B* and *R* are the luminance values of each pixel in the defined cell, *I*_MAX_ and *I*_MIN_ are the maximum and minimum of the diagonal values in the cell, respectively, and *w*_1_ and *w*_2_ are the weight coefficients, which represent the contribution of diagonal color components to the cell. The greater the contribution, the larger the weight that will be assigned to the components. According to the results of multiple tests, *w*_1_ = 0.6, *w*_2_ = 0.4 in our method. All the cells in raw data are processed in this way.

In this procedure, the diagonal elements in each cell, one of which is two values of G and the others are B and R values, are combined to generate the target intensity which contains the essential information of the cell. Thus, the reconstruction operation making full use of abundant color information in raw data is conducive to enhancing the matching accuracy. Moreover, it reduces time complexity effectively as well, without the procedure of demosaicing for acquiring a full color image with traditional methods.

### 2.3. Parallel and Integrated Framework

**Figure 2:**
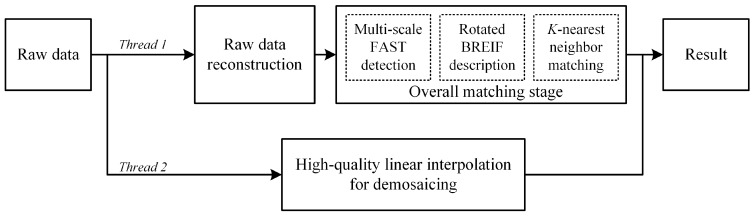
Flow chart of PIMR.

With the focus on efficiency of computation, in our methodology, two cores in a multi-core processor are used to parallel the procedure of matching and imaging. As shown in Figure 2, thread 1 is responsible for handling the raw data matching, and thread 2 performs demosaicing to get a full color image with high quality. Eventually, the final results are obtained by merging the outcomes from the two different threads.

When handling the raw data matching, first of all, we preprocess it using the raw data reconstruction step whose detailed implementation is in Section 2.2. Since the intensity information of the pixels after reconstruction has a similar data form to gray images, we adopt the multi-scale FAST detector and the rotated BRIEF descriptor in ORB to complete feature detection and description for raw data, which achieves high robustness to general image transformations, including image blur, rotation, scale and illuminance change, and is of fast speed as well [13]. For the part of feature matching, we first find the *k*-nearest neighbors in the sensed image to the keypoints in the reference image via brute-force matching, and then employ the ratio test explained by Lowe [7] to select the best one from the *k* matches. The three parts mentioned above, *i.e.*, the feature detection, description and matching, are collectively referred to as the overall matching stage in this paper. Moreover, note that the similarity between descriptors is measured by Hamming distance [10], which can be computed rapidly via a bitwise exclusive or (XOR) operation followed by a bit count. However, as the development of the multimedia instruction set of the central processing unit (CPU) is fast, it is more effective to calculate the number of bits set to 1 using the newer instructions on modern CPUs compared with the previous bit count operation. Hence, the population count (POPCNT) instruction, which is part of SSE4.2, is applied to our method for speeding up feature matching [24].

We use Malvar’s algorithm for raw data demosacing [25]. The method computes an interpolation using a bilinear technique, computes a gradient correction term, and linearly combines the interpolation and the correction term to produce a corrected, high-quality interpolation of a missing color value at a pixel. A gradient-correction gain is also used to control how much correction is applied, and the gain parameters are computed by a Wiener approach and they contribute to the coefficients of the linear filter in the method. The approach outperforms most nonlinear and linear demosaicing algorithms with a reduced computational complexity. Due to the high image quality and low computational cost, the method is considered the major demosaicing approach in industrial digital cameras [26].

## 3. Performance Evaluation

### 3.1. Experimental Details

We evaluate PIMR using a well-known dataset, *i.e.*, the Affine Covariant Features dataset introduced by Mikolajczyk and Schimid [27].

**Figure 3:**
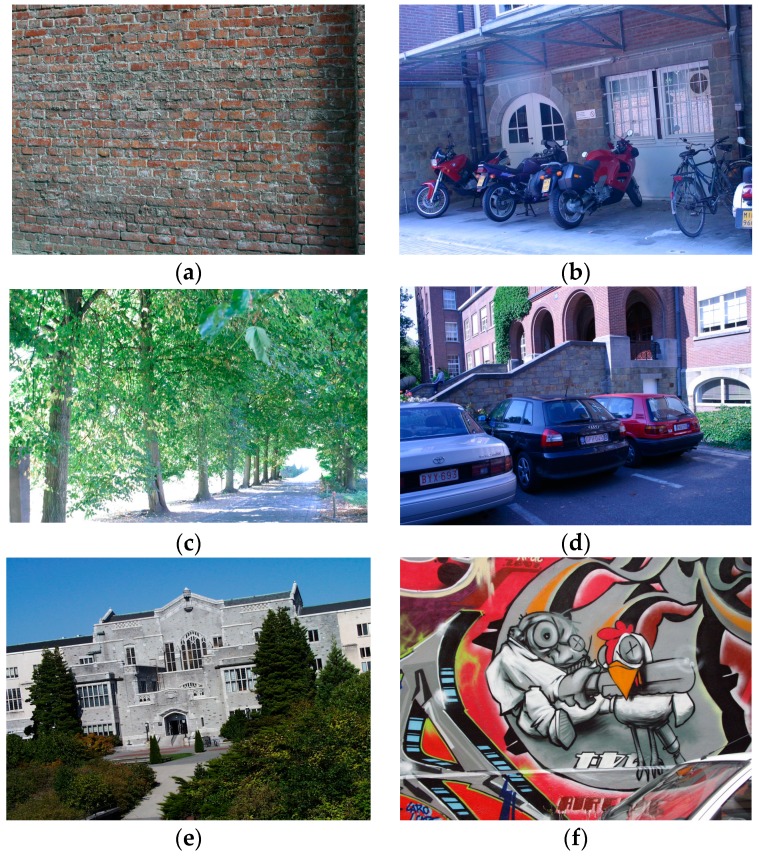
Image sequences from the Affine Covariant Features dataset. (**a**) wall; (**b**) bikes; (**c**) trees; (**d**) leuven; (**e**) UBC; (**f**) graffiti.

It should be noted that our approach directly processes raw data captured by the digital camera without demosaicing. For this reason, we need to extract the corresponding color information at each pixel location from the original images in the chosen dataset, in accordance with the GBRG mode of the Bayer pattern. The new dataset, being made up of a series of raw data, mainly contains the following six sequences: wall (view point change), bikes and trees (image blur), leuven (illumination change), University of British Columbia (UBC) (JPEG compression), and graffiti (rotation). Each sequence consists of six sets of raw data just as six images, sorted in order of a gradually increasing degree of distortions with respect to the first image with the exception of the graffiti sequence. We take the raw data of image 1 in each sequence as the reference data, and match the reference one against the remaining ones, yielding five matching pairs per sequence (1|2, 1|3, 1|4, 1|5 and 1|6). Figure 3 shows the first image in the original six sequences from the standard dataset.

Since our work aims at realizing robust and fast feature detection, description and matching for raw data, we assess not only the accuracy but also the time-cost, comparing our PIMR with the state-of-the-art algorithms, including BRIEF, ORB, BRISK, and FREAK.

We compute 1000 keypoints on five scales per raw data with a scaling factor of 1.3 using PIMR. In order to ensure a valid and fair assessment, the full color image processed by the BRIEF, ORB, BRISK and FREAK algorithms is demosaiced from raw data using the same interpolation approach as PIMR. It must be emphasized that we combine the BRIEF descriptor with the CenSurE detector, and the multi-scale adaptive and generic corner detection based on the accelerated segment test (AGAST) detector proposed in BRISK with the FREAK descriptor, based on the settings in reference [10,16]. The implementation of these methods is built with OpenCV 2.4.8 which provides a common two-dimensional (2D) feature interface.

### 3.2. Accuracy

**Figure 4:**
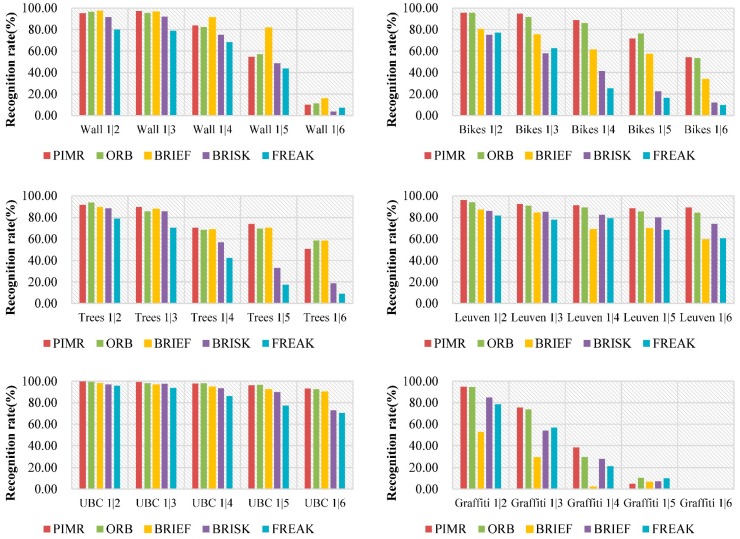
Recognition rates achieved by PIMR, ORB, BRIEF, BRISK and FREAK.

We use the recognition rate, namely the number of correct matches *versus* the number of total good matches, as the evaluation criterion. The results are shown in Figure 4. Each group of five bars with different colors represents the recognition rates of PIMR, ORB, BRIEF, BRISK and FREAK, respectively.

On the basis of these plots, we make the following observations. In general, our algorithm performs well for all test sequences, yielding comparable recognition accuracy with the state-of-the-art algorithms, and even outperforms them in most cases. There are only a few exceptions. For example, in the wall sequence, BRIEF achieves slightly higher precision than our methods, but in the other five sequences, PIMR outperforms the other algorithms.

### 3.3. Time-Cost

Table 1 shows the time-cost of the bikes 1|3 employing different algorithms, measured on an Intel Core i7 processor at 3.4 GHz, in seconds. Since there is no raw data construction step in traditional methods, and the demosaicing is processed in parallel with the other stages within the proposed approach, the procedures marked with dashes in the table involve no extra time. Note that the time is averaged over 50 runs.

Considering the total time listed in the last column of Table 1, PIMR is about 1.4× faster than ORB and 7.1× faster than FREAK. In PIMR, the time-cost of demosaicing is excluded from the total time, as its demosaicing stage is faster than the sum of the other two stages. One potential reason for the high computational cost in BRISK and FREAK is the application of the scale-space FAST-based detector [15] which promises coping with the scale invariance.

**Table 1:** Times of matching for the bikes image 1 and 3.

Methods	Demosaicing (s)	Raw Data Reconstruction (s)	Overall Matching (s)	Total (s)
ORB	0.009	-	0.040	0.049
BRIEF	0.009	-	0.049	0.058
BRISK	0.009	-	0.225	0.234
FREAK	0.009	-	0.231	0.240
PIMR	-	0.004	0.030	0.034

### 3.4. Matching Samples with PIMR

**Figure 5:**
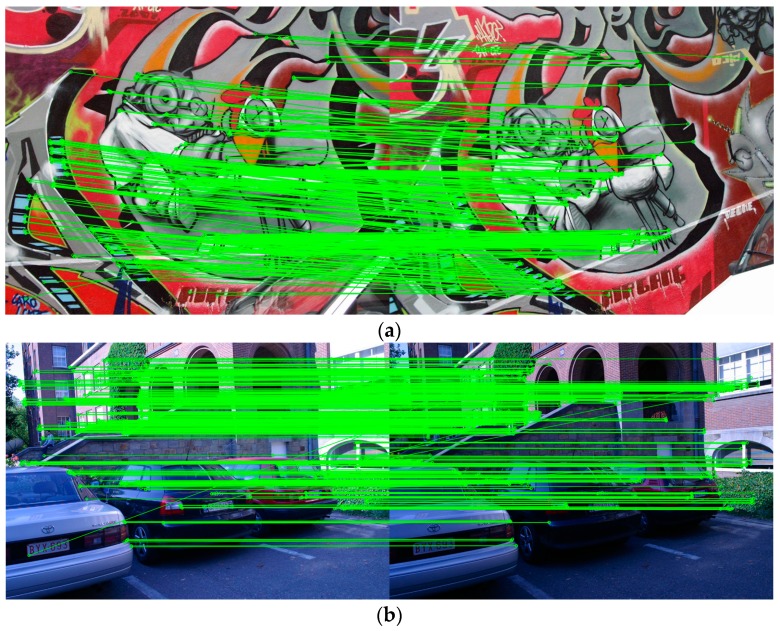
Matching samples using the PIMR. (**a**) The matching result of the graffiti sequence; (**b**) The matching result of the leuven sequence.

Extensive evaluations have been made for PIMR above, and we also provide some matching samples with the chosen dataset in this part. It is considered that the invariance of rotation, scale and illuminance is the overriding concern in image matching algorithms. Thereby, we present the matching results of the graffiti (rotation and scale change) and leuven (illumination change) sequences to further demonstrate the robustness of the proposed approach. Figure 5a shows the matching result of the graffiti sequence (1|2), and Figure 5b shows the matching result of the leuven sequence (1|2). The keypoints connected by green lines indicate keypoint correspondences, namely valid matches. It can be seen that the method PIMR can acquire sufficient robust matches with few outliers.

## 4. Conclusions

We have presented a parallel and integrated matching algorithm for raw data named PIMR, mainly to speed up whole image matching and to enhance the robustness as well. In most cases, it achieves better performance for most image transformations than current approaches, with a fairly low computational cost. Furthermore, our work is not only basic research in the field of rapid image matching, but also a preliminary study with respect to a method for processing raw data. In the future, an ultra-light-weight local binary descriptor will be studied further.
